# Paediatric pneumonia research priorities in the context of COVID-19: An eDelphi study

**DOI:** 10.7189/jogh.12.09001

**Published:** 2022-02-26

**Authors:** Carina King, Kevin Baker, Sol Richardson, Alexa Wharton-Smith, Ayobami A Bakare, Fyezah Jehan, Mohammod Jobayer Chisti, Heather Zar, Shally Awasthi, Helen Smith, Leith Greenslade, Shamim A Qazi

**Affiliations:** 1 Department of Global Public Health, Karolinska Institutet, Stockholm, Sweden; 2 Institute for Global Health, University College London, London, UK; 3 Malaria Consortium, London, UK; 4 Department of Community Medicine, University College Hospital Ibadan, Ibadan, Nigeria; 5 Department of Paediatrics and Child Health, The Aga Khan University, Karachi, Pakistan; 6 Nutrition and Clinical Services Division, International Centre for Diarrhoeal Disease Research Bangladesh (icddr,b), Dhaka, Bangladesh; 7 Department of Paediatrics and Child Health and SA-MRC Unit on Child & Adolescent Health, University of Cape Town, Cape Town, South Africa; 8 Department of Paediatrics, King George’s Medical University, Lucknow, India; 9 Consultant, International Health Consulting Services Ltd, UK; 10 Every Breath Counts Coalition, New York, New York, USA; 11 Consultant, Retired staff World Health Organization, Geneva, Switzerland

## Abstract

**Background:**

Pneumonia remains the leading cause of infectious deaths in children under-five globally. We update the research priorities for childhood pneumonia in the context of the COVID-19 pandemic and explore whether previous priorities have been addressed.

**Methods:**

We conducted an eDelphi study from November 2019 to June 2021. Experts were invited to take part, targeting balance by: gender, profession, and high (HIC) and low- and middle-income countries (LMIC). We followed a three-stage approach: 1. Collating questions, using a list published in 2011 and adding newly posed topics; 2. Narrowing down, through participant scoring on importance and whether they had been answered; 3. Ranking of retained topics. Topics were categorized into: prevent and protect, diagnosis, treatment and cross-cutting.

**Results:**

Overall 379 experts were identified, and 108 took part. We started with 83 topics, and 81 further general and 40 COVID-19 specific topics were proposed. In the final ranking 101 topics were retained, and the highest ranked was to *“explore interventions to prevent neonatal pneumonia”.* Among the top 20 topics, epidemiological research and intervention evaluation was commonly prioritized, followed by the operational and implementation research. Two COVID-19 related questions were ranked within the top 20. There were clear differences in priorities between HIC and LMIC respondents, and academics vs non-academics.

**Conclusions:**

Operational research on health system capacities, and evaluating optimized delivery of existing treatments, diagnostics and case management approaches are needed. This list should act as a catalyst for collaborative research, especially to meet the top priority in preventing neonatal pneumonia, and encourage multi-disciplinary partnerships.

Pneumonia remains the leading cause of infectious death among children under-five years of age globally, and since 2010 progress to reduce these deaths has lagged behind diarrhoea, measles and malaria. Pneumonia has been described as a neglected disease of poverty and a “*global cause without champions*” [1], with a disproportionate amount of funding is allocated to pneumonia research compared with other infections such as HIV/AIDS and malaria [2]. With an estimated 672 000 pneumonia deaths in children under-five in 2019 [3], major investments in research, implementation, health systems and advocacy are still needed to meet Sustainable Development Goal (SDG) 3.2 – to end preventable deaths of newborns and children, with national targets of fewer than 12 neonatal deaths per 1000 live births and under-5 mortality of 25 per 1000 live births [4].

Given funding gaps for pneumonia, well-conducted and relevant research targeting the major barriers to further mortality reductions is urgently needed [5]. A paediatric pneumonia research priority-setting exercise was first conducted in 2008, with four areas of focus identified: 1) better understanding of aetiology to guide vaccine targets and antimicrobial therapy; 2) study the pathogenesis to improve case management; 3) develop surveillance tools and new diagnostics; and 4) evaluate the impact of prevention and case management strategies on mortality and equity [6]. A subsequent exercise coordinated by the World Health Organisation (WHO) published a research priority list for pneumonia in 2011 with 45 leading childhood pneumonia researchers suggesting more than 500 research ideas [7]. These were refined into 158 research questions spanning a broad spectrum of multi-disciplinary research from basic science to health policies and systems.

Since then, there have been significant advances in addressing some priority areas. Pneumococcal conjugate vaccines (PCV) have been introduced in 148 countries, and *Haemophillus influenza B* vaccine coverage is at 70% [8]. Much progress has also been made in development of novel RSV preventive interventions, with over 60 candidates in pre-clinical or clinical trials [9]. Prevention and management of paediatric HIV has been strengthened, with reductions in perinatally infected children [10]. Socioeconomic improvements have also led to better health for children and families. Innovations in commercially available medical technologies, such as portable lung ultrasound, pulse oximetry and multi-modal devices [11-15], provide new diagnostic approaches but raise implementation questions. And seminal epidemiological studies [16,17], clinical trials for oxygen therapies [18-20], and antibiotic treatment regimens [21-23] have reinforced challenges in optimal case management.

However, the COVID-19 pandemic has had major impacts on health and health systems. While children have been largely spared from direct clinical effects in the first waves, indirect effects, such as interruptions to routine immunization [24], the loss of caregivers [25], and shifting health services and resources towards managing the substantial caseloads in adults, may have longer term implications. We therefore set-out to update the research priorities for childhood pneumonia in the context of the COVID-19 pandemic and explore whether previous priority areas have been addressed. The ultimate goal is to increase investment in the areas of pneumonia research that have the greatest potential to accelerate mortality reductions and enable LMICs to achieve the SDGs.

## METHODS

We conducted an electronic Delphi (eDelphi) research prioritisation, from November 2019 to June 2021. There were four rounds of web-surveys, with a pause in the study from March 2020 - January 2021 due to the COVID-19 pandemic. This work was conducted by the Every Breath Counts Coalition (EBC) Research Group. EBC is a public-private coalition of over 50 organizations working together to support governments to reduce pneumonia deaths in low- and middle-income countries (LMICs) [26].

We followed a three-stage eDelphi approach adapted from Okoli & Pawlowski (2004) [27]. This method was selected given its pragmatic nature, requiring only a nominal time commitment from expert participants, and ability to work across a geographically dispersed group. This methodology has been widely used for health research and policy priority-setting, with its online adaptation notable for its participant retention and satisfaction [28]. Anonymity aims to ensure the process is not dominated by one expert and there is less pressure to conform to the group position [29].

### Expert selection

‘Experts’ were defined’ as clinicians, researchers, technical partners and implementers, and industry and government employees who have actively worked in the field of childhood pneumonia within the last five-years. There is no established prerequisite number of participants needed for an eDelphi study [30]. Considering potential non-response and attrition throughout survey rounds and desire for wide geographical reach, we aimed to assemble a list with a minimum of 300 experts, and wanted to achieve a minimum of 50 respondents per round.

A list was collated by CK, KB and AWS, from the following sources: the proposed list of invited participants for the first Global Pneumonia Forum hosted in Barcelona, January 2020; authors from seminal paediatric pneumonia publications from 2015-2019; leads of pneumonia research groups; heads of key bilateral, international and local non-governmental organisations (NGO) pneumonia programmes in high-burden countries; Ministry of Health Integrated Management of Childhood Illnesses (IMCI) and Acute Respiratory Infection (ARI) programme coordinators; pharmaceutical and medical device manufacturers. Information on their current affiliation, gender, country of residence and email contact was extracted, where publicly available. Participants without a valid email contact were excluded.

The list was refined to achieve equal representation from multilateral, bilateral, national governments, NGOs, academic or research institutions, donors, and private companies. In addition, we sought equal gender distribution, a minimum of 50% from LMICs, and a minimum of 50% from the 10 highest burden countries (Chad, Nigeria, Angola, Niger, Somalia, Mali, DRC, Afghanistan, Pakistan, Ethiopia). All participants were then emailed, explaining the study and asking them to opt-in to participate in subsequent rounds of the study.

### Stage 1: Collating questions

The *first stage* involved collating an initial list of research topics. We used the full 158 research questions published by Rudan et al. in 2011 [7], and added new topics which had been posed by the EBC steering group during an initial planning in March 2019. KB, AWS, SM and CK went through the list and re-worded, combined and refined the questions to reduce ambiguity and minimise duplications. We categorised each question into four over-arching themes, to align with the Global Action Plan for Pneumonia and Diarrhoea (GAPPD) framework [31]: prevent and protect (vaccination, care-seeking, nutrition), diagnose (case management, point of care tests, devices), treat (antibiotics, antivirals, oxygen) and cross-cutting (epidemiology, policy, health systems).

During the first web-survey, experts were able to provide up to three additional research questions under each theme. Data collection was stopped due to the pandemic in March 2020, due to concerns about time and availability of participants, and the anticipated effect on research priorities. In the second web-survey, sent in January 2021, we asked experts to provide up to three COVID-19 related research questions. At this time point, vaccines had been approved for adults but not children and the Delta variant was not yet widespread. Questions which were related solely to the COVID-19 pandemic with no association to paediatric pneumonia (eg, adult immune responses) were excluded. We followed the same process of combining, refinement and categorisation into themes, and included all newly proposed eligible questions in the next web-survey round.

### Stage 2: Narrowing down

The *second stage* narrowed down the research topics to retain those that were classified as both important and unanswered. Experts were asked to score each research topic on a scale of 1-5 for its importance and whether it has been answered (1 = not at all; 2 = somewhat; 3 = fairly; 4 = very; 5 = extremely). We dichotomised both questions, with “unanswered” (not at all or somewhat answered) and “answered” (fairly, very and extremely), and “important” (very or extremely important) and “not important” (fairly, somewhat and not at all). Research topics which had ≥50% classified as both unanswered and important were retained for the next round. Respondents were given the option to select which themes they responded to.

Narrowing down was done over three web-survey rounds, including: 1. the refined Rudan et al list [7]; 2. newly proposed questions and retained questions from round one; 3. newly proposed COVID-19 related questions. The results from rounds two and three were then combined to form a single list of unanswered and important questions for ranking. The survey was conducted using SmartSurvey (SmartSurvey, Gloucestershire, UK), and the link was emailed to respondents. Three reminder emails were sent for each survey round; in addition, KB and CK presented at the January 2020 Global Pneumonia Forum to recruit participants, and shared the link from personal, EBC and Malaria Consortium Twitter accounts.

### Stage 3: Ranking

The *third stage* was ranking the research questions which were deemed unanswered and important by the expert group. Experts were asked to give a numerical ranking, with 1 as the most important, for each research topic within themes. One ranking round was conducted. The link was emailed to experts, with three reminder emails. In addition, CK and KB sent personalised direct emails to participants from Rounds 2 and 3 to increase participation.

### Analysis

The final ranking was calculated using a re-scaled mean of the rank, with a lower mean indicating a higher priority. As the four themes had different numbers of questions, we re-scaled each set of questions to be scored from 0-25 to allow comparison and an overall rank. We conducted a sensitivity analysis using the inverse mean to rank the research priorities. This takes the reciprocal of the ranking value (ie, one divided by the rank) and therefore higher ranked responses are given more weight in the mean. Kendall’s W coefficient of concordance was calculated to explore the level of consensus reached in the ranking, with 0 indicating no consensus, and >0.7 considered strong agreement [32]. We further classified the final ranked list into types of research, using the ‘4Ds’ approach: description, delivery, development and discovery [33].

We stratified rankings by the income level of respondent’s country of current residence (HIC vs LMIC), and by academic vs non-academic respondents. Kendall’s tau rank correlation coefficient was used to measure the ordinal association between overall ranks by these sub-groups. We then conducted Mann-Whitney U tests to compare statistically different rankings given for each research priority by respondents based in HICs and LMICs, and by academic vs non-academic respondents. Raw data was exported from SmartSurvey as Excel files, and analysis and data processing was conducted using Stata SE14 (StataCorp, College Station, TX, USA).

### Ethics

The protocol was reviewed and given a favourable opinion by the Liverpool School of Tropical Medicine research ethics committee (reference: 19-081). Participants were provided study information and asked to electronically confirm consent at the start of each web-survey.

## RESULTS

### Participants

We compiled a list of 379 pneumonia experts, with email contacts found for 370 (97%). A further 31 participants were recruited from advertising through the various EBC platforms, and overall, 108 different experts took part. **Table 1** summarises the participants across the three prioritisation and the final ranking rounds.

**Table 1:** Expert participants across the rounds of prioritisation and ranking

		Original invite list (n = 370)*	Round 1 (n = 60)	Round 2 (n = 74)	Round 3 (n = 32)	Round 4 (n = 49)
Gender	Male	185 (50%)	23 (38%)	41 (55%)	18 (56%)	29 (59%)
	Female	181 (48%)	31 (512%)	33 (45%)	14 (44%)	20 (41%)
	Missing	4 (1%)	6 (10%)	-	-	-
Institution	Research	148 (40%)	26 (43%)	32 (43%)	18 (56%)	28 (57%)
	NGO/iNGO	108 (29%)	16 (27%)	31 (42%)	9 (28%)	14 (29%)
	Government	69 (19%)	6 (10%)	4 (5%)	2 (6%)	4 (8%)
	Private	45 (12%)	6 (10%)	4 (5%)	3 (9%)	3 (6%)
	Missing	-	6 (10%)	3 (4%)	-	-
Region†	HIC	196 (53%)	42 (70%)	46 (62%)	22 (69%)	35 (71%)
	UMIC	34 (9%)	1 (2%)	1 (1%)	-	1 (2%)
	LMIC	88 (24%)	13 (22%)	14 (19%)	8 (25%)	11 (23%)
	LIC	52 (14%)	4 (7%)	13 (18%)	2 (6%)	2 (4%)

*The 9 experts where no email could be found are excluded.

†This represents the region where the respondent was residing, not their nationality or the region in which their work is focused. Income status is based on the World Bank 2020 classification. HIC: high-income country; UMIC: upper-middle income country; LMIC: lower-middle income country; LIC: low-income country.

**Figure 1:**
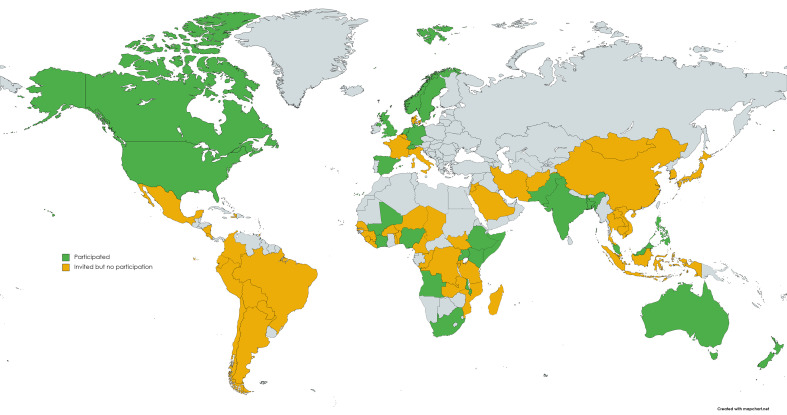
Map showing countries represented in the list of invited experts and the location of those who participated.

We started with an equal gender balance, 40% of experts within research institutes and 53% based in HICs. In the final ranking round, 59% of respondents were male, 57% were academics and 71% were based in HICs. There were no respondents from South and Central America (**Figure 1**), and the highest number of participants were based in the USA (n = 26), UK (n = 21) and Uganda (n = 10).

### Prioritisation

**Figure 2:**
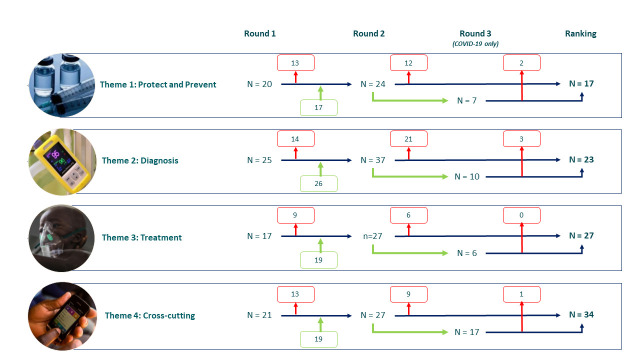
Research topics added and retained across the prioritisation rounds, by themes. Red boxes indicate the research topics which did not score highly enough to be retained. Green boxes indicate the new research topics suggested by respondents and were included.

The first survey round included 83 research topics, with balance across the four themes ranging from 25 questions (30%) in diagnosis to 17 (21%) in treatment themes (**Figure 2**). From Round 1, an additional 81 topics were added, and from Round 2 a further 40 COVID-19 specific topics were included. The final ranking had 101 retained topics, with an increase in cross-cutting questions (n = 34, 34%). Notably, only 15% (n = 6/40) of the COVID-19 questions were dropped in Round 3 as these questions were mostly scored as “not answered”.

Taking the top 10 list from Rudan et al (2011) [7], two topics were retained following Round 1 (**Table 2**), all scoring high on importance, but not reaching the 50% threshold for remaining unanswered. Of the two that remained, they were ranked as the overall 2^nd^ and 6^th^ highest priorities – **Table 3**.

**Table 2:** List of top 10 priorities from Rudan et al (2011) [7] and their current scoring

Rank	Original research topic *[minor edits]*	Revised research topic*	Theme	Importance	Unanswered	Retained
1	Study the main barriers to health care seeking and health care access for children with pneumonia in different contexts and settings in developing countries	*Explore care-seeking behaviour for childhood respiratory illnesses*	Cross-cutting	87%	42%	Dropped in Round 1
2	Identify the key risk factors predisposing to the development of severe pneumonia and identify children who require hospitalisation†	*Identify diagnostic markers of severity and their utility in identifying children who merit referral for pneumonia care*	Diagnosis	95%	38%	Dropped in Round 2
		*Identify clinical signs and simple laboratory tests that predict poor treatment outcomes*	Diagnosis	94%	48%	Dropped in Round 1
		*Assess the effectiveness of existing WHO treatment algorithms and guidelines on preventing pneumonia-related deaths, unnecessary referrals and unnecessary antibiotic use*	Diagnosis	86%	30%	Dropped in Round 2
3	Study the main barriers to increased coverage of available vaccines - Hib vaccine and pneumococcal vaccine - in different contexts and settings	*Explore vaccine acceptability / uptake / hesitancy / compliance among both patient and clinicians, health care worker populations*	Prevent & protect	79%	38%	Dropped in Round 1
5	Study the main barriers to increasing demand for/compliance with vaccination with available vaccines in different contexts and settings – for measles and pertussis vaccines, Hib vaccine and pneumococcal vaccine					
4	Study whether the coverage by antibiotic treatment can be greatly expanded in safe and effective way if it was administered by community health workers		Treatment	83%	36%	Dropped in Round 1
6	Assess the effectiveness of new conjugate pneumococcal vaccines in the reduction of childhood pneumonia morbidity and mortality in different settings	*Assess the efficacy and effectiveness of new vaccines in reduction of childhood pneumonia morbidity and mortality in different populations and settings, such as pregnant women*	Prevent & protect	75%	63%	**Yes – ranked 6^th^ overall**
7	Identify the key bacterial and non-bacterial pathogens associated with childhood pneumonia morbidity and mortality at the global level in HIV and non-HIV-infected children		Diagnosis	86%	15%	Dropped in Round 1
8	*Assess if* community volunteers be trained to adequately assess, recognise danger signs, refer and treat ARI?		Cross-cutting	84%	35%	Dropped in Round 1
9	Study the capacity of health systems worldwide to correctly diagnose and manage childhood pneumonia, and obstacles to correct diagnosis and case management in developing country settings		Cross-cutting	94%	54%	**Yes – ranked 2^nd^ overall**
10	Identify *and evaluate* cost reduction mechanisms for conjugate vaccines (eg, regional purchasing consortia, private public partnerships and novel funding mechanisms)		Prevent & protect	83%	38%	Dropped in Round 1

*Blank if the question was retained with only minor language edits.

†Questions relating to risk factors such as malnutrition, HIV and pollution were already included, therefore this question was split to explore different components of clinical signs of severity and case management.

**Table 3:** Top 5 ranked research topics within each theme

Research topic		Overall rank		Research type	Scaled Mean*	Inverse mean ranking†
**Prevent & protect:**						
1	Explore interventions to prevent neonatal pneumonia	1	Description		7.21	1
2	Assess RSV vaccine efficacy, effectiveness, cost-effectiveness and proxy surrogates of protection	3	Description		8.25	2
3	Assess the efficacy and effectiveness of new vaccines in reducing childhood pneumonia morbidity and mortality in different populations and settings, such as in pregnant women	6	Description		8.77	9
4	Study the barriers to reducing indoor air pollution, including reducing smoking, increase emission cleanliness of household fuel, reducing cost for complete combustion of biomass fuels	20	Description		10.42	10
5	Develop low cost, conjugate/combination vaccines or multiple respiratory viral antigens (Human MPV, Influenza and Parainfluenza)	27	Discovery		11.03	5
**Diagnosis:**						
1	Develop inexpensive and rapid point-of care diagnostic and aetiological tests that differentiate bacterial, viral (incl. RSV) and malaria infections that are reliable in in community settings and at facilities in children and young infants	4	Discovery		8.72	1
2	Implementation research to identify best ways of integrating pulse oximetry and oxygen into IMCI and other existing protocols	5	Delivery		8.75	2
3	Identify clinical signs, simple laboratory tests and biomarkers that predict poor treatment outcomes and need for further care	7	Description		8.80	3
4	Evaluate the effect of pulse oximetry introduction on care practices, referral uptake, time to treatment and outcomes in primary and secondary health care settings	8	Description		8.83	5
5	Assess the role and challenges in using pulse oximetry at the community level, particularly in populations where severe anaemia	19	Description		10.19	12
**Treatment:**						
1	Identify health system capacity, and the main barriers to providing oxygen in health facilities	9	Delivery		8.84	2
2	Develop improved oxygen concentrators, eg, in terms of reduction in size, reliability, affordability, length of lifetime without maintenance, ability to run independent of electricity supply, accessibility/more easily deliverable in both community setting and clinical practice	10	Development		8.99	6
3	Evaluate situations where antibiotics may be appropriately withheld to avoid unnecessary antibiotic use, including for non-severe pneumonia	11	Description		9.12	1
4	Explore alternative antibiotic treatment regimens for pneumonia, including short course once daily regimes	12	Description		9.32	5
5	Assess the cost-effectiveness of oxygen, including different systems, at different levels of the health system	13	Description		9.35	4
**Cross-cutting:**						
1	Study the capacity of health systems worldwide to correctly diagnose and manage childhood pneumonia, and obstacles to correct diagnosis and case management in low-resource settings	2	Delivery		7.56	1
2	What is the impact of the COVID-19 pandemic on access to child health services, including for pneumonia in LMIC contexts?	14	Description		9.55	7
3	Assess the quality of care provided to children with pneumonia and/or hypoxemia at community, primary, and secondary levels of care (including iCCM, IMCI, and emergency triage and treatment)	16	Description		10.00	3
4	Develop validated risk prediction models across a range of resource settings	17	Development		10.04	4
5	Investigate the long-term effects of COVID-19 pneumonia infections on child health and development	18	Description		10.16	10

*The ranking was re-scaled so each theme used the same range from 1-25; a lower value indicates a higher rank (ie, a value of 1 would mean all respondents ranking this as the highest priority).

†The inverse mean was calculated from the reciprocal of the scaled ranking, with a range from 1-0.04; a higher value indicates a higher rank. All Kendall-W coefficients of concordance were <0.1.

### Ranking

**Table 3** presents the top five ranked research topics within each of the four themes, and their overall ranking. The highest ranked topic overall was to *“explore interventions to prevent neonatal pneumonia*”, with a mean rank of 7.21 out of 25. Only one of the topics ranked in the top five for the theme would not have been in the top 20 overall – on developing low-cost conjugate vaccines for multiple respiratory pathogens. Of the 34 COVID-19 specific questions, two were ranked in the top 20, with these topics generally scoring as lower priority. While all research types were represented in the list, description (ie, observational epidemiology and intervention evaluation, n = 13/20) was the most common. Notably in the treatment theme, none of the top priorities involved discovery of new treatments or therapies. The full list of topics and their rank are presented in Table S1 in the **Online Supplementary Document**. There was good agreement with the inverse mean rankings, but concordance in the ranking between respondents overall was poor.

In the analyses by income-status of respondent’s country of residence, there was no overlap in the top five priorities. Further, three of the top five priorities from LMIC respondents were not in the top 20 overall priorities (**Table 4**). There were also clear differences in priorities from academic respondents compared with non-academics, with the 2^nd^ priority around health systems capacity being ranked 18^th^ amongst academics (Table S1 in the **Online Supplementary Document**). Overall questions ranked higher by HIC respondents received higher ranks from respondents in LMICs, with a moderate positive correlation (ρ = 0.329, *P* < 0.001). A similar correlation was seen for academic vs non-academic respondents (ρ = 0.320, *P* < 0.001).

**Table 4:** Top five priorities stratified by respondent country income-level*

Rank	High-income country respondents (n = 35)				Low- and middle-income country respondents (n = 14)			
**Research topic**	**Theme**	**Research type**	**Overall rank (LMIC rank)**	**Research topic**	**Theme**	**Research type**	**Overall rank (HIC rank)**
1	Explore interventions to prevent neonatal pneumonia	Prevent & protect	Description	1 (18)	Develop low cost, conjugate/combination vaccines or multiple respiratory viral antigens (Human MPV, Influenza and Parainfluenza)	Prevent & protect	Discovery	27 (71)
2	Study the capacity of health systems worldwide to correctly diagnose and manage childhood pneumonia, and obstacles to correct diagnosis and case management in low-resource settings	Cross-cutting	Delivery	2 (10)	Identify the health systems capacity, and the main barriers to providing oxygen in health facilities	Treat	Delivery	9 (17)
3	Assess RSV vaccine efficacy, effectiveness, cost-effectiveness and proxy surrogates of protection	Prevent & protect	Description	3 (8)	Develop strategies for differentiating bronchiolitis from bacterial pneumonia, and subsequent bronchiolitis care pathways for low and middle income settings	Diagnose	Development	28 (51)
4	Evaluate the effect of pulse oximetry introduction on care practices, referral uptake, time to treatment and outcomes in primary and secondary health care settings	Diagnosis	Description	8 (28)	Understand the epidemiology of pneumonia severity and mortality in children presenting with COVID-19 symptoms	Cross-cutting	Description	33 (58)
5	Evaluate situations where antibiotics may be appropriately withheld to avoid unnecessary antibiotic use, including for non-severe pneumonia	Treat	Description	11 (31)	Identify clinical signs, simple laboratory tests and biomarkers that predict poor treatment outcomes and need for further care	Diagnose	Description	7 (11)

HIC – high-income country, LMIC – low and middle-income country

*Note: respondents chose which themes to respond to and therefore the means were calculated using different denominators, leading to some research topics being ranked lower than expected in sub-group analyses.

The results of the Mann-Whitney U tests showed HIC respondents were significantly more likely (*P* < 0.05) to give higher ranks to three questions (overall ranked questions: 1, 23 and 54 – Table S1 in the in the **Online Supplementary Document**), while LMIC respondents ranked four items higher (overall ranked questions: 27, 89, 94 and 95). Academic respondents ranked two questions higher (overall ranked questions: 3 and 93), and non-academics ranked six items higher (overall ranked questions: 2, 14, 47, 49, 59 and 94).

## DISCUSSION

In this eDelphi study for childhood pneumonia research priorities in the context of the COVID-19 pandemic, the highest priority topic was to “*explore interventions to prevent neonatal pneumonia*”. The opinions expressed by the participants appear to corroborate the notion that the biggest challenge in addressing pneumonia effectively is to increase access to, and refine existing effective solutions for, those populations with the heaviest burden of mortality. However, priorities were not consistent between HIC and LMIC, and between academic and non-academic respondents.

That the top priority is for research on neonates reflects the mortality burden, with 47% of deaths in under-fives occurring in this group;(34) although this proportion is not consistent across regions. While neonatal mortality has declined by 19% since 2010, this is much slower than the 36% decline seen in children aged 1-59 months [34]. A newborn health research priority-setting exercise from 2014 included several questions relating to etection and management of neonatal infections (eg, home-based injectable antibiotics to treat sepsis) [35], while social, behavioural and community engagement priorities have highlighted the need to understand drivers of health care worker performance and community access [36]. Given the difficulty in distinguishing sepsis and pneumonia and the multiple calls for systems-focused approaches, it is important that research on neonatal survival strategies is collaborative. However, LMIC respondents only ranked this 18^th^ and non-academics as the 6^th^ priority. Given nearly three-quarters of respondents in the ranking were based in HICs, this priority is skewed towards a high-income perspective.

This reflects wider differences seen between the views of LMIC and HIC respondents, and similarly between academics and non-academic respondents (including Ministry of Health, NGOs, multilaterals and private companies). While a broad correlation in rankings was indicated by the positive Kendall’s tau rank correlation coefficients, there were significant differences in the likelihood of respondents giving different rankings for a number of individual questions. This disconnect between the academic community, implementers and policy makers has been previously highlighted in a prioritisation exercise [36], and reflects the different orientations of each. The top five priorities from non-academics were focused on more practical questions around current capacity, service delivery and quality of care; conversely academic priorities targeted new interventions and refinement of existing case management and technologies. Further, the majority of funding for health research in LMICs is awarded to HIC institutions [37], exacerbating the gap between locally identified needs and LMIC academic interests. While funding innovation is important, these crucial discrepancies add to the argument that in-country long-term operational research capacity and implementation science needs to be equally supported and rewarded [38,39].

Multiple research topics were retained and ranked highly on refining differential diagnosis and antibiotic protocols. This reflects the persistent challenge of how to improve antibiotic targeting to reduce over-treatment of viral infections, maintaining access, and minimising the threat of antibiotic resistance. Notably, a focus on alternative antiviral treatments was not seen. Similarly, questions on key pneumonia determinants and mortality risks was largely absent. While indoor air pollution featured 20^th^ (and 73^rd^), malnutrition 25^th^, and HIV/AIDS 86^th^, topics addressing women’s empowerment, poverty and crowding did not appear on the final list. These are well-established and enumerated risks (ie, ‘answered’) [40], and broader societal and political issues (ie, not limited to paediatric pneumonia), and may have been de-prioritised as a result. Therefore, it is important to reiterate that knowledge generation alone has been insufficient, and that linking evidence to advocacy, implementation and policy still needs to be strengthened. Including policy makers early in the research process and understanding their needs could improve this gap [41,42]. Despite deliberate efforts to engage Ministry of Health perspectives in this process we were unsuccessful; dissemination plans should specifically target local stakeholders, including this group.

Adding COVID-19 related topics resulted in the inclusion of a large number of novel research areas, however, only two were retained in the top 20 priorities. This may reflect the challenges in tackling COVID-19, such as weak health systems and limited oxygen capacity, already being relevant for managing paediatric pneumonia. It may also reflect the relatively low direct impact on children. However, we are in an uncertain epidemiological period for paediatric respiratory aetiology, with decreased influenza transmission and paediatric respiratory hospital admissions, coinciding with COVID-19 control measures [43-47]. Low infant exposure to common respiratory pathogens during the pandemic may result in subsequent outbreaks, as is already being reported for RSV [48]. Therefore, epidemiological research questions should ensure the local COVID-19 context is considered, and if/how control strategies have mitigated or exacerbated the paediatric pneumonia burden.

Diagnosis and treatment priorities had a health systems focus, suggesting tools to manage and treat pneumonia exist, but quality implementation and targeting for different groups at scale remains challenging. When we compare our results with the Rudan et al (2011) priorities [7], most of the basic epidemiological questions have been addressed. This is supported by multiple, complex, multi-site studies being completed (eg, PERCH [16] or then Drakenstein Child Health study [49]), or expected to report soon (eg, HAPIN [50]). This suggests larger, longer term, collaborative platforms have been effective in providing definitive evidence. Prospective evaluation of research investments in pneumonia is required, to understand which research funding approaches are most impactful, and monitor progress against priority areas.

We had three major limitations in our study, first, all the study information and surveys were provided English only, which may have impacted expert’s ability and willingness to participate from regions such as Central and South America where we had no respondents. Second, engagement from LMIC practitioners was hard to maintain, in particular we did not have sufficient representation from the 10 highest burden countries. We attempted to mitigate this with focused recruitment through a variety of channels such as the 2020 Global Pneumonia Forum and UNICEF/Save the Children face-to-face events. Ultimately, this justified our decision not to conduct a second ranking round to establish whether consensus could be improved, as we were concerned engagement would become less representative and the study period had already been prolonged. Third, while our use of a consensus method with a large expert group, from diverse specialties and geographical locations should provide legitimacy, the eDelphi method has limitations as it is expert-opinion based. We did not include community members or patients, and are limited by the composition of the panel. The initial content presented to the panel was aligned with WHO initiatives, and developed by a diverse specialist team, but the possibility remains that alternative methods and respondents would have produced different priorities. We propose follow-up activities are conducted in high burden pneumonia countries, with stakeholder consultations to adopt national level pneumonia research priorities. We hope this would further address the differences between HIC and LMIC priorities in this work.

## CONCLUSIONS

These child pneumonia research priorities should be taken as the established list in the countdown to 2030, and influence global clinicians, researchers, and implementers as well as research funders. This exercise found an urgent need for more investment in pneumonia innovation and discovery, in operational research and in the application of complex systems thinking. We hope this acts a catalyst for collaborations across research topics, disciplines and organisational boundaries and challenge the many Every Breath Counts coalition members and supporters (including governments, NGOs, regional paediatric and pulmonary forums, companies, research institutes, and donors), to ensure that the top-ranked priorities are funded and then translated into action to accelerate national and global progress in achieving the SDG goals and targets.

## Additional material


Online Supplementary Document
