# Supplementary material for: Paediatric pneumonia research priorities in the context of COVID-19: An eDelphi study
Source: J Glob Health. 2022 Feb 26;12:09001. doi: 10.7189/jogh.12.09001 (PMC8874896; doi:10.7189/jogh.12.09001)
Supplement: Online Supplementary Document [file jogh-12-09001-s001.pdf]

Appendix 1: Overall summary of prioritisation and ranking for all retained research topics

Overall rank	Research Topic	Theme	Research type	Scaled mean*	Sub-group rankings				
					Within theme	HIC	LMIC	Academic	Non-academic
1	Explore interventions to prevent neonatal pneumonia	Prevent	Description	7.21	1	1	18	2	6
2	Study the capacity of health systems worldwide to correctly diagnose and manage childhood pneumonia, and obstacles to correct diagnosis and case management in low-resource settings	Cross-cutting	Delivery	7.56	1	2	10	18	1
3	Assess RSV vaccine efficacy, effectiveness, cost-effectiveness and proxy surrogates of protection	Prevent	Description	8.25	2	3	8	1	38
4	Develop inexpensive and rapid point-of care diagnostic and aetiological tests that differentiate bacterial, viral (incl. RSV) and malaria infections that are reliable in in community settings and at facilities in children and young infants	Diagnose	Discovery	8.72	1	7	15	22	3
5	Implementation research to identify best ways of integrating pulse oximetry and oxygen into IMCI and other existing protocols	Diagnose	Delivery	8.75	2	6	17	5	17
6	Assess the efficacy and effectiveness of new vaccines in reducing childhood pneumonia morbidity and mortality in different populations and settings, such as in pregnant women	Prevent	Description	8.77	3	13	6	7	10
7	Identify clinical signs, simple lab tests and biomarkers that predict poor treatment outcomes and need for further care	Diagnose	Description	8.80	3	11	5	4	25
8	Evaluate the effect of pulse oximetry introduction on care practices, referral uptake, time to treatment and outcomes in primary and secondary health care settings	Diagnose	Description	8.83	4	4	28	23	4
9	Identify the health systems capacity, and the main barriers to providing oxygen in health facilities	Treat	Delivery	8.84	1	17	2	14	5
10	Develop improved oxygen concentrators, e.g. in terms of reduction in size, reliability, affordability, length of lifetime without maintenance, ability to run independent of electricity supply, accessibility/more easily deliverable in both community setting and clinical practice	Treat	Development	8.99	2	16	7	10	8
11	Evaluate situations where antibiotics may be appropriately withheld to avoid unnecessary antibiotic use, including for non-severe pneumonia	Treat	Description	9.12	3	5	31	3	34
12	Explore alternative antibiotic treatment regimens for pneumonia, including short course once daily regimes	Treat	Description	9.32	4	14	12	8	18
13	Assess the cost-effectiveness of oxygen, including different systems, at different levels of the health system	Treat	Description	9.35	5	15	13	19	7
14	*What is the impact of the COVID-19 pandemic on access to child health services, including for pneumonia in LMIC contexts?*	Cross-cutting	Description	9.55	2	9	33	17	9
15	Explore use of medical oxygen for paediatric pneumonia cases (challenges, gaps, facilitators, uptake)	Treat	Delivery	9.85	6	8	56	26	11
16	Assess the quality of care provided to children with pneumonia and/or hypoxemia at community, primary, and secondary levels of care (including iCCM, IMCI, and emergency triage and treatment)	Cross-cutting	Description	10.00	3	19	21	60	2

17	Develop validated risk prediction models across a range of resource settings	Cross-cutting	Development	10.04	4	10	48	6	44
18	*Investigate the long-term effects of COVID-19 pneumonia infections on child health and development*	Cross-cutting	Description	10.16	5	20	20	9	37
19	Assess the role and challenges in using pulse oximetry at the community level, particularly in populations where severe anaemia	Diagnose	Description	10.19	5	18	40	11	28
20	Study the barriers to reducing indoor air pollution, including reducing smoking, increase emission cleanliness of household fuel, reducing cost for complete combustion of biomass fuels	Prevent	Description	10.42	4	21	25	34	13
21	Develop better models and tools to estimate burden of pneumonia disease and burden of pneumonia mortality	Cross-cutting	Development	10.57	6	31	14	13	42
22	*What effect has the COVID-19 pandemic had on efforts to reduce the burden of childhood pneumonia (e.g. health service unavailability) plus child education and development?*	Cross-cutting	Description	10.64	7	23	41	27	27
23	Assess how improvements in terms of universal health coverage improves pneumonia outcomes	Cross-cutting	Description	10.92	8	12	88	28	30
24	Explore low cost cheap emergency transport oxygen systems for referrals	Treat	Discovery	10.94	7	25	43	38	22
25	Develop newer tools and algorithms for the diagnosis of pneumonia in children with malnutrition?	Diagnose	Development	10.97	6	27	34	39	20
26	*Can combination point-of-care tests, using biomarkers, screen for and diagnose viral, bacterial and COVID-19 pneumonia?*	Diagnose	Description	10.97	7	26	36	40	21
27	Develop low cost, conjugate/combination vaccines or multiple respiratory viral antigens (Human MPV, Influenza and Parainfluenza)	Prevent	Discovery	11.03	5	71	1	49	14
28	Develop strategies for differentiating bronchiolitis from bacterial pneumonia, and subsequent bronchiolitis care pathways for low and middle income settings	Diagnose	Development	11.05	8	51	3	12	59
29	Development, test and implement algorithms to identify children at low risk of bacterial pneumonia and who are not treated with antibiotics	Diagnose	Development	11.18	9	24	57	21	48
30	Investigate the epidemiology of recurrent pneumonia episodes	Cross-cutting	Description	11.27	9	33	37	25	47
31	*Is there any change in paediatric pneumonia aetiology after the emergence of COVID-19?*	Cross-cutting	Description	11.31	10	35	35	15	66
32	Optimise community-based oxygen therapy treatment of lower respiratory infections and assess its effectiveness	Treat	Delivery	11.32	8	30	45	48	24
33	*Understand the epidemiology of pneumonia severity and mortality in children presenting with COVID-19 symptoms*	Cross-cutting	Description	11.39	11	58	4	46	23
34	Development of new antimicrobial agents that could overcome bacterial resistance	Treat	Discovery	11.43	9	40	19	32	41
35	Develop vaccines resistant to heat (i.e. that do not require a cold chain)	Prevent	Discovery	11.46	6	22	70	24	58
36	Explore biomarkers of treatment response in pneumonia	Treat	Description	11.62	10	37	42	16	67

37	*Explore whether lung damage caused by COVID-19 infections (including asymptomatic/mild cases), lead to risk of subsequent respiratory disease*	Cross-cutting	Description	11.68	12	29	61	30	56
38	Develop low cost point of care tools to accurately assess correct vaccination status	Prevent	Discovery	11.68	7	46	24	41	32
39	Assess barriers to the identification of children needing referral, and the compliance with and completion of referrals in children with pneumonia	Diagnose	Description	11.73	10	41	29	20	73
40	Evaluate national policy, financial, and supply chain commitments and barriers to supply chains for child-friendly amoxicillin formulation and oxygen services	Treat	Delivery	11.76	11	32	59	29	55
41	Understand the epidemiology of pneumonia severity and mortality in children with multiple acute infections (e.g. diarrhoea/sepsis) compared to those with pneumonia alone	Cross-cutting	Description	11.80	13	28	72	33	57
42	Optimise pneumonia treatment stopping rules and early discharge rules	Treat	Development	12.14	12	42	46	31	68
43	Measure the effect of improved workforce numbers on child health outcomes, including pneumonia	Cross-cutting	Description	12.15	14	36	71	72	16
44	Explore the cost-effectiveness of pulse oximetry different levels of the health system, including outpatient settings	Diagnose	Description	12.18	11	38	62	57	36
45	Develop an inexpensive and rapid point of care test to determine antibiotic sensitivity in bacterial pneumonias	Diagnose	Discovery	12.18	12	67	11	59	33
46	*How has the prioritisation of child health in national and local policy changed due to COVID-19, where adults are most affected?*	Cross-cutting	Delivery	12.20	15	55	27	58	35
47	Describe the distribution of oxygen accessible at primary health centers as an emergency when children with acute symptoms are detected	Treat	Description	12.24	13	54	38	88	12
48	*What benefits has the COVID-19 pandemic had for efforts to reduce the burden of childhood pneumonia (e.g. vaccine access, drug supplies, pulse oximetry, oxygen)?*	Cross-cutting	Description	12.26	16	45	50	44	49
49	Identify systems capacity to improve the quality of supportive care for children with pneumonia in health facilities and reduce nosocomial infections	Cross-cutting	Delivery	12.27	17	39	55	77	15
50	Assess the barriers to effective pneumonia case management using a health systems approach to consider factors such as supply chain, repair and replace as well as clinical and resource factors	Cross-cutting	Delivery	12.38	18	44	54	69	26
51	Develop effective supportive treatment packages for viral respiratory infections (e.g. low cost monoclonal antibodies for RSV)	Treat	Discovery	12.54	14	81	9	42	63
52	Understand the impact of different health financing and governance mechanisms for pneumonia prevention	Cross-cutting	Delivery	12.54	19	47	58	47	60
53	Establish the determinants of healthy lung development, including lower respiratory tract infections, nutrition and climate	Prevent	Description	12.74	8	43	73	43	72
54	Can new severity marker-based triage tests be used for improved risk stratification, and how do they compare to current triage tools?	Diagnose	Discovery	12.79	13	34	98	35	82

55	*Can automated diagnostic tools (including lung ultrasound and CXR) aid differential diagnosis of bacterial, viral and COVID-19 pneumonias?*	Diagnose	Describe	12.87	14	72	30	54	64
56	Conduct cost-effectiveness studies for the treatment of both non-severe and severe pneumonia	Treat	Delivery	12.89	15	48	82	79	31
57	*Determine the all cause pneumonia burden pre and post COVID-19*	Cross-cutting	Description	12.99	20	77	32	53	74
58	Explore approaches to reducing the cost of pulse oximeters and making them more available to general public	Diagnose	Development	13.00	15	74	39	52	76
59	Identify and evaluate cost reduction mechanisms for continuous supply of conjugate vaccines (e.g. regional purchasing consortia, private public partnerships and novel funding mechanisms)	Prevent	Delivery	13.02	9	49	83	87	19
60	*Determine the burden of and risk factors for severe respiratory COVID-19 illness in children*	Cross-cutting	Description	13.10	21	66	51	50	78
61	Refine the current verbal autopsy tool for pneumonia death diagnosis, taking advantage of post-mortem mortality surveillance platforms	Cross-cutting	Development	13.13	22	52	84	62	65
62	Explore the chronology of mixed infections in pneumonia and their role in susceptibility, morbidity and mortality	Prevent	Description	13.14	10	56	66	37	90
63	*How has the incidence and diagnosis of pneumonia, including severe cases, changed for children during the COVID-19 pandemic?*	Cross-cutting	Description	13.16	23	87	16	61	69
64	Study the epidemiology of pneumonia sequelae, including cardiac complications	Cross-cutting	Description	13.30	24	62	64	36	93
65	*Does COVID-19 predispose children to concomitant bacterial or other viral infections or recurrent pneumonias after initial infection?*	Prevent	Description	13.32	11	73	52	66	61
66	*How can governments and facilities utilise oxygen procurement and training for COVID-19 to support for other primary healthcare needs, including severe pneumonia?*	Treat	Delivery	13.34	16	76	47	73	51
67	*What is the optimal home management strategy for children with a bacterial respiratory and COVID-19 co-infection?*	Treat	Delivery	13.37	17	64	67	84	40
68	Identify new approaches to maintenance of equipment used to treat pneumonia, such as oxygen	Treat	Development	13.41	18	53	89	51	79
69	*Assess COVID-19 and bacterial pneumonia co-infection related morbidities in children*	Cross-cutting	Description	13.43	25	63	74	76	45
70	*Amongst children with WHO defined pneumonia, what proportion are COVID-19 positive, viral, bacterial or co-infections?*	Cross-cutting	Description	13.46	26	78	49	65	75
71	Understand the role of the microbiome in pneumonia susceptibility and infection	Cross-cutting	Description	13.47	27	60	78	45	88
72	Evaluate the role of digital auscultation in identifying diagnosing pneumonia, differentiating lung and cough sounds, and differentiating bronchiolitis	Diagnose	Description	13.49	16	70	60	75	52
73	Summarise data on the critical concentration of pollutants that cause pneumonia	Prevent	Description	13.52	12	59	79	86	29
74	*What are the opportunities for re-deployment and improved device availability and coverage for oxygen commodities post COVID-19?*	Treat	Delivery	13.54	19	65	76	68	70

75	Link research on financial sustainability for different interventions at CHW, community and health system levels	Cross-cutting	Delivery	13.60	28	83	44	85	39
76	Develop pneumonia treatment strategies for children aged 5–14	Treat	Development	13.64	20	50	100	80	53
77	*What should the recommended treatment for COVID-19 pneumonia be, including severe cases, in the paediatric population?*	Treat	Description	13.65	21	69	77	89	43
78	Evaluate the performance and potential role of artificial intelligence and machine learning in diagnosing pneumonia in children	Diagnose	Discovery	13.83	17	61	91	63	84
79	Study caregiver/patient compliance with treatment, including both oxygen and antibiotics	Treat	Description	13.89	22	75	80	55	89
80	Evaluate interventions to improve provider counselling of preventive and promotive behaviours during sick child visits and/or antenatal, vaccine and postnatal visits	Prevent	Description	13.93	13	57	95	67	81
81	*Understand COVID-19 vaccine responses in children*	Prevent	Description	13.93	14	79	75	64	87
82	Determine how best to assess new automated pneumonia-diagnosis technology (e.g. multi-modals), including accuracy, demand, usability and appropriateness, at different levels of the health system	Diagnose	Discovery	13.98	18	68	90	90	46
83	*Understand the ethics and impact of COVID-19 vaccination amongst children to protect communities*	Prevent	Description	14.22	15	80	85	70	85
84	Investigate if responsive monitoring charts improve care practices and outcomes for children with pneumonia	Treat	Description	14.25	23	84	68	92	62
85	Develop and utilise fine-level (sub district) mapping of pneumonia burden by age group and risk factors	Cross-cutting	Delivery	14.30	29	82	87	82	71
86	Are there any interactions between COVID-19 and co-infections, such as HIV and TB, and other pneumonia pathogens?	Cross-cutting	Description	14.36	30	86	65	71	86
87	Determine the cost-effectiveness of different diagnostic approaches, including different devices, at different levels of the health system	Diagnose	Delivery	14.36	19	85	69	83	77
88	Do we need a revised pneumonia case management guidelines (e.g. IMCI and iCCM) during the COVID-19 pandemic?	Diagnose	Description	14.51	20	89	63	93	50
89	Evaluate the use of nebulization / bronchodilation among children with respiratory symptoms	Treat	Description	14.81	24	93	22	74	92
90	Describe the political economy of pneumonia in order to determine how to make governments/MoH/Donors/Agencies care about pneumonia in a context of competing priorities and dedicate time and resources to tackling it	Cross-cutting	Delivery	15.00	31	88	92	91	80
91	Understand childhood pneumonia aetiology in the presence of maternal NCDs such as diabetes, asthma, COPD	Cross-cutting	Description	15.14	32	92	53	81	94
92	What is the prevalence of COVID-19 virus in the airways of the general paediatric population?	Cross-cutting	Description	15.29	33	91	86	78	95
93	Assess the prevalence of counterfeit antibiotics and implications	Treat	Description	15.49	25	90	97	56	101
94	What is the impact of pneumococcal vaccines on COVID-19 infections?	Prevent	Description	15.71	16	98	26	99	54

95	Does improved pneumonia identification preserve hospital capacity for COVID-19?	Cross-cutting	Delivery	16.01	34	100	23	94	83
96	Does pneumonia increase children's susceptibility to symptomatic and severe COVID-19?	Prevent	Description	16.55	17	94	94	96	91
97	In areas with high COVID-19 infection rates, is there a benefit to treating all children who present with respiratory symptoms with first line antibiotics (e.g. Amox - DT)?	Treat	Description	17.05	26	99	81	95	97
98	Has COVID-19 impacted the irrational use of antibiotics for paediatric respiratory infections?	Treat	Description	17.23	27	96	93	98	96
99	Clinical symptoms and profile of COVID-19 pneumonia in pregnant mothers	Diagnose	Description	17.30	21	95	96	97	99
100	Can a differential diagnosis between COVID-19 pneumonia and bacterial pneumonia be made at community level (e.g. using iCCM)?	Diagnose	Description	17.86	22	97	101	100	100
101	Determine the ability to distinguish between COVID-19 and a diagnosis of other causes of childhood pneumonia using clinical signs	Diagnose	Description	18.00	23	101	99	101	98

*A lower value indicates a higher ranking; the values can range between 1 – 25.
